# The Dissector; or, Practical Surgical Anatomy

**Published:** 1851-08

**Authors:** 


Art. VI. — *The Dissector; or, Practical Surgical Anatomy.* By Erasmus Wilson, author of a "System of Human Anatomy." With one hundred and fifteen illustrations. Edited by Paul B. Goddard, M.D. A new and improved edition. Philadelphia: Blanchard & Lea. 1851.

This work is supposed to be especially useful to students, as it undoubtedly is, but it has higher claims to professional respect and favor. In addition to its many good qualities as a mere dissecting assistant, its department of surgical anatomy is very full and complete, and must prove an excellent aid to the physician who may be called, in a sudden emergency, to perform a critical surgical operation.

The wood cuts of this volume are among the very best that we have seen in an American work, and the volume is highly creditable both to editor and publishers. B.

